# Protective Effects of Transforming Growth Factor β2 in Intestinal Epithelial Cells by Regulation of Proteins Associated with Stress and Endotoxin Responses

**DOI:** 10.1371/journal.pone.0117608

**Published:** 2015-02-10

**Authors:** Duc Ninh Nguyen, Pingping Jiang, Susanne Jacobsen, Per T. Sangild, Emøke Bendixen, Dereck E. W. Chatterton

**Affiliations:** 1 Department of Food Science, Faculty of Science, University of Copenhagen, Frederiksberg, Denmark; 2 Department of Nutrition, Exercise and Sports, Faculty of Science, University of Copenhagen, Frederiksberg, Denmark; 3 Department of Systems Biology, Technical University of Denmark, Lyngby, Denmark; 4 Department of Molecular Biology and Genetics, Aarhus University, Aarhus, Denmark; Vanderbilt University, UNITED STATES

## Abstract

Transforming growth factor (TGF)-β2 is an important anti-inflammatory protein in milk and colostrum. TGF-β2 supplementation appears to reduce gut inflammatory diseases in early life, such as necrotizing enterocolitis (NEC) in young mice. However, the molecular mechanisms by which TGF-β2 protects immature intestinal epithelial cells (IECs) remain to be more clearly elucidated before interventions in infants can be considered. Porcine IECs PsIc1 were treated with TGF-β2 and/or lipopolysaccharide (LPS), and changes in the cellular proteome were subsequently analyzed using two-dimensional gel electrophoresis-MS and LC-MS-based proteomics. TGF-β2 alone induced the differential expression of 13 proteins and the majority of the identified proteins were associated with stress responses, TGF-β and Toll-like receptor 4 signaling cascades. In particular, a series of heat shock proteins had similar differential trends as previously shown in the intestine of NEC-resistant preterm pigs and young mice. Furthermore, LC-MS-based proteomics and Western blot analyses revealed 20 differentially expressed proteins following treatment with TGF-β2 in LPS-challenged IECs. Thirteen of these proteins were associated with stress response pathways, among which five proteins were altered by LPS and restored by TGF-β2, whereas six were differentially expressed only by TGF-β2 in LPS-challenged IECs. Based on previously reported biological functions, these patterns indicate the anti-stress and anti-inflammatory effects of TGF-β2 in IECs. We conclude that TGF-β2 of dietary or endogenous origin may regulate the IEC responses against LPS stimuli, thereby supporting cellular homeostasis and innate immunity in response to bacterial colonization, and the first enteral feeding in early life.

## Introduction

Transforming growth factor β2 (TGF-β2) is an important growth factor present in human and bovine milk (0.1–5.3 and 13–1150 ng/mL, respectively). TGF-β2 may promote intestinal immune responses and gut functions, such as the intestinal adaptation to bacterial colonization and oral food intake, in newborn infants [1,2]. However, immediately after birth, the endogenous synthesis of TGF-β2 is negligible in the intestine of immature neonates [3]. This deficiency of TGF-β2 may partly account for intestinal disorders, for instance the high incidence of necrotizing enterocolitis (NEC) in formula-fed preterm infants [4,5]. TGF-β2 supplementation of enteral diets has been shown to protect against inflammatory diseases such as NEC [6] and intestinal bowel disease [7] in mice. On the other hand, in a murine model, the activation of Toll-like receptor 4 (TLR-4) in intestinal epithelial cells (IECs) by lipopolysaccharide (LPS) derived from Gram-negative bacteria plays a central role in NEC progression through the inhibition of IEC migration and proliferation, thereby leading to IEC apoptosis and NEC development [8]. These observations suggest that the synergy of TGF-β2 and LPS may play important roles in modulating intestinal diseases such as NEC.

We have established a clinically relevant preterm pig model to investigate the effects of various diets on the pathogenesis of intestinal disorders including NEC [9]. Through proteomic analysis, we have repeatedly identified differentially expressed heat shock proteins (HSPs), particularly elevated HSP70s and reduced HSP90B1 in the intestine of NEC-resistant preterm pigs, compared with conventionally reared and formula-fed pigs, which are susceptible to NEC development [10–14]. In mice, the specific expression of HSP70s in IECs is pivotal in decreasing TLR-4 signaling, thereby protecting against NEC [8]. Thus, it is highly interesting to elucidate whether these HSPs show similar behaviors in piglet IECs.

Using this preterm pig model of NEC, we have shown that the enteral feeding of bovine colostrum, which is abundant in TGF-β2, decreases NEC incidence and down-regulates intestinal inflammatory mediators, compared with formula feeding not containing TGF-β2 [9]. The supplementation of TGF-β2 to formula has not yet been tested in infants or piglets, partly due to the high cost of this peptide. Mechanistic studies performed in an *in-vitro* porcine IEC model under a controlled environment, without confounding factors such as bacterial colonization and enteral feeding, are important to confirm the protective effects of TGF-β2, before studies in preterm pigs and infants can be considered. We have therefore used a porcine IEC cell line (PsIc1), derived from the intestinal crypts of a six-month old pig as a not fully differentiated and immature cell model, to investigate cellular mechanisms and support the rationale for *in-vivo* studies. TGF-β2 has already been shown to decrease inflammatory cytokine secretion in both PsIc1 cells [15] and other types of IECs [16]. However, it remains unknown whether TGF-β2 protects IECs by inducing differential protein expression in the IEC proteome, and whether these trends are similar to that found previously with the pattern of HSP regulation in the intestine of preterm pigs.

In the present study, we hypothesized that TGF-β2, at a dose similar to its level in mature human milk, stimulates changes in the proteome of porcine IECs to protect the cells against stress induced by pathogens and thereby maintains cellular homeostasis. The effects of TGF-β2 on the proteome of naïve and inflamed IECs induced by LPS were profiled using gel-based and LC-MS-based proteomics in PsIc1 cells.

## Materials and Methods

### Cell culture

Porcine IECs (PsIc1) from 6 month-old pigs were established as previously reported [17] and were provided by Bionutritech (Lunel, France). At passages 5–25, the cells were cultured in T75 flasks using advanced DMEM medium supplemented with 2% heat-inactivated fetal bovine serum, 40 U/mL penicillin, 40 μg/mL streptomycin and 2 mM glutamax (all materials for cell culture were from Life Technologies, Nærum, Denmark) at 37°C and 5% CO_2_.

### Gel-based proteomics of IECs treated by TGF-β2

PsIc1 cells, at 90–95% confluency, were treated with 3 ng/mL TGF-β2 (Santa Cruz Biotechnology, CA) in serum-free medium (TGF) or only serum-free medium without TGF-β2 (CON) for 24 h. Five replicates of the CON and TGF cells were harvested. Cellular protein extraction, purification, two-dimensional gel electrophoresis (2DE) and MS were performed as previously described with minor modifications [11]. Briefly, the cells from each replicate were lyzed in a cocktail buffer containing 1% (v/v) Triton X-100 with the addition of 1.5% (v/v) protease inhibitor (Sigma-Aldrich, Germany). The lysate was cleaned up using the TCA incubation method [11], then re-suspended in a buffer containing 7 M Urea, 2 M Thiourea, 4% CHAPS and 2 mM tributylphosphine (TBP, BioRad, Hercules, CA). The protein concentration was determined using the 2D Quant Kit (GE Healthcare, Buckinghamshire, UK).

In total, 10 gels were run (5 CON and 5 TGF). Briefly, 100 μg of extracted protein of one replicate was mixed with re-hydration buffer (9.5 M urea, 4% CHAPS, 2 mM TBP, 0.2% carrier ampholyte 3–10 NL) to a total volume of 300 μL, and applied onto a 17-cm ReadyStrip IPG strip (3–10 NL, BioRad) for isoelectric focusing (IEF) on a PROTEAN IEF system (BioRad) according to the following program: the voltage was linearly increased up to 250 V in 30 min, then linearly increased up to 10,000 V in 3 h, finally holding at 10,000 V until reaching 70,000 Vh. SDS-PAGE of the focused gel strips was performed on 1.0 mm-thick 12.5% SDS-PAGE gels after a two-step equilibration with DTT and iodoacetamide. Following electrophoresis, the gels were stained with SYPRO Ruby Total Protein Stain (Bio-Rad) according to the manufacturer’s instructions. The stained gels were scanned on a Typhoon scanner (GE Healthcare) and analyzed by the 2D gel analysis software Image Master Platinum 2D 5.0 (GE Healthcare). The matched spots were automatically assigned with numbers. The expression levels of the spots were calculated in percentage volume (% Vol) and exported for statistical analysis. Differentially expressed spots between CON and TGF groups (P < 0.05) were treated by in-gel trypsin digestion as previously described [11]. The tryptic peptide mixtures were analyzed using an Ultraflex II MALDI-TOF/TOF mass spectrometer (Bruker Daltonics, Bremen, Germany). Generated mass spectra were used for protein identity searching against the NCBInr database with taxonomy limited to *Mammalia* using MASCOT 2.0 software integrated with BioTools 3.1 (Bruker Daltonics). A Mascot score of ≥ 74 for peptide mass fingerprinting and ≥ 40 for MS/MS was considered significant. The information for the identified proteins including protein name, GenInfo identifier, protein score, expression levels, searched pI and relative molecular weight was recorded.

For validation, extracted proteins from the CON and TGF cells were resolved by SDS-PAGE, transferred onto PVDF membranes (Life Technologies), and probed with specific primary antibodies as previously described [18]. The band density of targeted proteins was detected by chemiluminescence. Anti-HSP60, HSPA5, HSPA8, HSP90B1 antibodies (Abcam, Cambridge, UK) were used. The protein expression levels were normalized to the level of β-actin (Santa Cruz, CA, USA).

### LC-MS/MS proteomics of LPS-challenged IECs treated with TGF-β2

To further investigate the effects of TGF-β2 on the cellular proteome of LPS-challenged IECs, the proteome of naïve cells in serum-free medium (CON), and cells treated with 1 μg/mL LPS alone (LPS) or 3 ng/mL TGF-β2 and 1 μg/mL LPS (TGF+LPS) were compared. In this study, a high-throughput approach, compared with the gel-based system, was adopted by utilizing LC-based proteomics with iTRAQ (isobaric tag for relative and absolute quantitation) labeling. Each group was performed in triplicate in a similar manner as described for gel-based proteomics. The cells were washed twice with cold PBS, scraped and centrifuged at 2500 × g for 5 min for cell collection. The cells were stored at -80°C until further analysis. Cell lysis was performed by sonication in TES buffer (10 mM Tris pH 7.6, 1 mM EDTA and 0.25 M sucrose) and the protein concentration was determined by the BCA protein assay (Thermo Scientific). A common reference sample was made by pooling equal amounts of protein from all samples in triplicates. This common reference was included in each of the 4-plexed samples as specified in Table 1. All protein extracts were precipitated in cold acetone; cysteines were reduced with DTT, and free cysteines were blocked with methyl methanethiosulfonate (MMTS) before performing trypsin digestion overnight. The resulting peptides were then labeled with specific iTRAQ tags according to the scheme in Table 1. This procedure was described previously in extensive details [19]. All 4-plexed samples were dried at room temperature and stored at -80°C until LC-MS/MS analysis.

**Table 1:** Design of iTRAQ labeling.

Isobaric tag	114	115	116	117
**Plex 1**	Ref	1A	2A	3A
**Plex 2**	Ref	1B	2B	4B
**Plex 3**	Ref	1C	3C	4C
**Plex 4**	Ref	2C	3B	4A

Ref: common reference sample appearing in all 4 plexes, generated by mixing equal protein amounts in all 12 samples (4 treatments in biological triplicates).

Sample 1, 2, 3, 4: Treatments of CON, TGF, LPS, TGF+LPS, respectively. As the TGF group was already investigated by gel-based proteomics, this group was removed from statistical comparison.

A, B, C: Biological triplicates

For MS analysis, the samples were re-suspended and dissolved in 0.03% formic acid (FA) in 5% acetonitrile. Protein (100 μg) from each 4-plexed sample was passed through strong cation exchange chromatography (SCX) followed by LC-MS/MS analysis as previously described [19]. Briefly, peptides eluted from the SCX chromatography were collected in one-minute fractions for 60 min and then pooled into 10 individual fractions. Each of these 10 fractions was analyzed individually, by loading onto an Agilent 1100 Series nanoflow HPLC combined with a Q-Star Elite mass spectrometer (Applied Biosystems, Life Technologies).

The raw data files were integrated into the Mascot search engine (Matrix Science). The mass accuracy of peptides and fragments was 15 ppm and 0.2 Da. The ion score expect cut-off was 0.005. The data files were searched using both NCBInr and UniProtKB databases for porcine sequences (*Sus scrofa*). The raw. mgf files for the 10 SCX fractions from each 4-plex sample were combined and searched as a single dataset. The search results for all individual 4-plexed samples were merged by MS Data Miner software (www.sourceforge.net/projects/msdataminer/) and exported as a combined Excel file for statistical analysis. Further details of database searching and full protein list used for statistics are presented in S1 Table. The mass spectrometry proteomic data have been deposited to the ProteomeXchange Consortium (http://proteomecentral.proteomexchange.org), from which all originial data files are freely available, under the dataset identifier number PXD001465.

To support the data of iTRAQ-coupled LC-MS/MS analysis, cells treated with LPS and/or TGF-β2 for 24 h were analyzed by Western blot to test the regulation of several additional proteins related to the identified stress-response proteins. The following proteins were measured: apoptosis-inducing factor (AIF), annexin A2, annexin A5, HSP27, HSP90, laminin receptor, calreticulin, protein kinase C alpha (PKC-α) and thioredoxin 5 (all antibodies from Abcam).

### Statistical analysis

The spot intensity from gel-based proteomics, relative abundance from LC-MS based proteomics as well as band intensity by densitometry from Western blot were aligned with treatments and imported into R (R Core Team, R Foundation for Statistical Computing, Vienna, Austria 2013) for statistical analyses. Each feature was fitted to a linear model with treatment as the fixed factor, and the significance of treatment was tested using ANOVA analysis of the fitted model. Differences in the mean of abundance among CON, LPS and TGF+LPS groups were further tested using “glht” function in the package ‘multcomp’ [20]. Validation of 2D-gel proteomics by Western blot was analyzed by densitometry and Student’s t-test (JMP, SAS Institute, Cary, NC). P-values were extracted from specific tests and a P < 0.05 was regarded as significance.

### Analysis of protein-protein interaction by bioinformatics

For the analysis of protein-protein interaction, identified proteins from both proteome analyses and Western blot were searched against the STRING database (string-db.org) with a confidence score ≥ 0.4. Only connected proteins were shown in the network.

## Results

### Differential IEC proteome induced by TGF-β2

**Fig 1:**
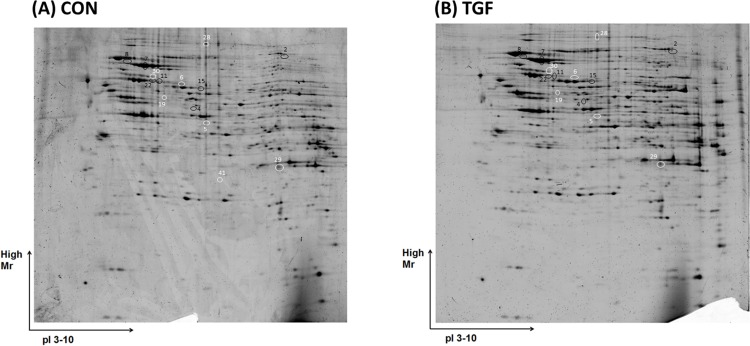
Representative SYPRO Ruby stained 2DE gels of cellular proteins of PsIc1 cells. (A): untreated cells (CON); (B): TGF-β2 treated cells (TGF). Black and white circles demonstrate protein spots with significant up-regulation and down-regulation, respectively, by TGF-β2.

The effects of TGF-β2 on the IEC proteome were investigated by gel-based proteomics. Fig. 1 shows representative 2DE gels for CON and TGF with protein spots detected in the range of pI 3–10 and relative molar mass (Mr) of 10–250 kDa. Forty-one spots were observed to be differentially expressed, and 13 protein spots were successfully identified by MS or MS/MS. The information for the identified proteins, including spot number, GeneInfo identifier, searched/apparent pI and Mr, expression levels, fold change and P-value, is summarized in Table 2. These 13 proteins were classified into four groups according to their reported biological functions: stress response, signal transduction, mRNA and DNA binding, as well as cytoskeleton remodeling and cell mobility. Five HSPs were identified, and four of which were up-regulated by TGF-β2 including three HSP70s (HSPA5, HSPA8 and HSPA9), and HSP60. Elevated levels of HSP70s and HSP60 suggest that TGF-β2 increases cellular protection against potential stress. The remaining detected HSP (HSP90B1) was down-regulated 2.5 fold by TGF-β2. Furthermore, four out of five proteins related to signal transduction, and RNA and DNA binding were down-regulated.

**Table 2:** Identified proteins differentially expressed by TGF-β2 in porcine IECs by gel-based proteomics.

No^1^	Protein name	GI Id ^2^	Score	pI (S/A) ^3^	Mr (S/A) ^3^	Expression levels		Ratio (TGF/CON)	P < 0.05
						CON	TGF		
**Stress response-proteins**									
7	Heat-shock cognate 71 kDa protein (HSPA8)	gi|471415065	104	5.8/5.4	56/73	2.16 ± 0.34	2.83 ± 0.14	+ 1.3	0.009
	Heat shock 70 kDa protein 9 (HSPA9)	gi|512959167	90	5.4/5.4	74/73								
11	Heat-shock 60 kDa protein (HSP60)	gi|359811347	136	5.6	61/61	0.06 ± 0.03	0.17 ± 0.07	+ 3.1	0.011
22	Heat-shock 60 kDa protein (HSP60)	gi|296439571	86	4.7/5.5	29/61	0.08 ± 0.01	0.12 ± 0.03	+ 1.5	0.027
8	Heat shock 70 kDa protein 5 (HSPA5)	gi|350579657	110	5.4/5.1	73/76	0.04 ± 0.01	0.08 ± 0.02	+ 2.2	0.010
28	Heat shock protein 90 kDa beta 1 (HSP90B1)	gi|301759325	78	4.8/6	93/98	0.14 ± 0.07	0.05 ± 0.02	- 2.5	0.046
**Signal transduction-related proteins**									
4	Somatotropin-like protein (GH1)	gi|390463200	78	6.17/6	247/52	0.01 ± 0.01	0.04 ± 0.01	+ 3.1	0.002
6	Protein phosphatase subunit G5PR (G5PR)	gi|48476968	76	5/5.8	50/58	0.07 ± 0.01	0.02 ± 0.02	- 3.5	0.004
29	Receptor of activated protein kinase C1- (RACK1)	gi|119574080	74	7.6/7.7	31/34	0.19 ± 0.05	0.12 ± 0.02	-1.5	0.049
**RNA and DNA binding proteins**									
5	Predicted: zinc finger protein 268-like (ZNF268)	gi|472392549	84	9.7/6.1	62/47	0.05 ± 0.01	0.01 ± 0.00	- 3.9	0.002
30	Heterogeneous nuclear ribonucleoprotein K	gi|392513715	55	5.5	51/58	0.05 ± 0.02	0.03 ± 0.01	- 1.9	0.049
**Cytoskeleton remodeling and cell mobility related proteins**									
15	p60 Katanin—like 2 protein (KATNAL2)	gi|470613275	84	7.2/6.2	63/59	0.08 ± 0.02	0.12 ± 0.02	+ 1.5	0.015
2	Keratin, type II cytoskeletal 1-like isoform 6	gi|297262447	85	8/8.2	63/73	0.02 ± 0.00	0.06 ± 0.01	+ 2.5	<0.001
41	Keratin, type II cytoskeletal 1 (KRT1)	gi|375314771	84	8.2/6.2	66/33	0.02 ± 0.01	0	+	

^1^ Spot number, as indicated in Fig. 1

^2^ Sequence identification number generated by GeneBank

^3^ Searched/apparent (S/A) isoelectric point (pI) and relative molar mass (Mr)

**Fig 2:**
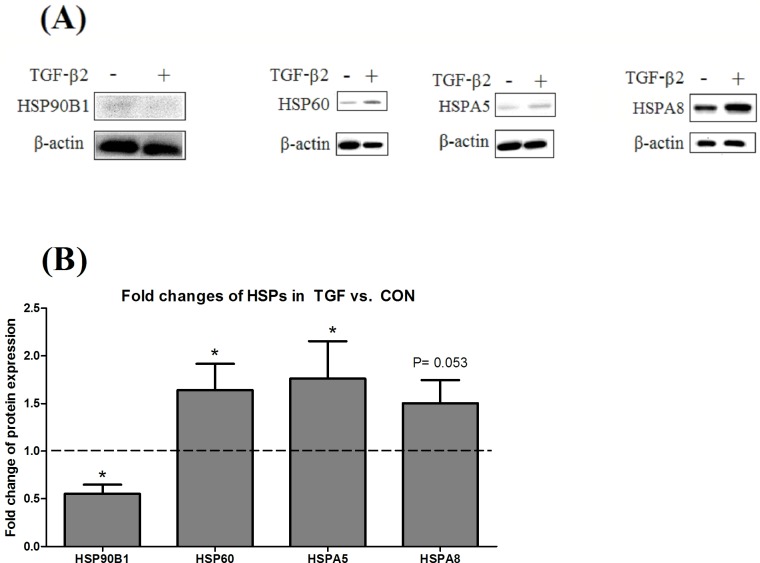
Western blot. Cross-validation of 2DE gel data by Western blot of HSP90B1, HSP60, HSPA5 and HSPA8 in control (CON) and TGF-β2 treated IECs (TGF). (A) Representative blots. (B) Fold-changes of HSPs in TGF, compared with CON analyzed by densitometry and Student’s t-test. Values were means ± SEM (n = 5 in each treatment). * P < 0.05, vs. CON group.

Cross-validation of the 2DE data by Western blot confirmed the up-regulation of HSP60 and HSPA5 and the down-regulation of HSP90B1 in TGF, compared with CON (P < 0.05). HSPA8 indicated a trend of increase in TGF compared with CON (Fig. 2).

### Differential LPS-challenged IEC proteome induced by TGF-β2

As stress-response proteins (particularly HSPs) were the major group differentially expressed by TGF-β2 in IECs, these proteins may play an important role in protecting the cells against inflammatory stressors such as LPS. Therefore, the effects of TGF-β2 on LPS-challenged IECs were further investigated by LC-MS based proteomics, a more sensitive and high-throughput approach than gel-based proteomics, to determine the molecular mechanisms by which TGF-β2 modulated the inflamed IECs. Ninety-three out of approximately 700 identified proteins appeared in at least 3 plexes and were selected for statistical analysis. Three groups of CON, LPS and TGF+LPS were statistically compared. A total of 15 proteins showed differences between the three treatment groups (P < 0.05, Table 3) and eight of these proteins were associated with stress response: glucose-regulated protein 58 (GRP58), protein disulfide isomerase (PDI), elongation factor 1-α1 (EEF1A1), cyclophilin A (CYPA), HSPA8, nucleolin, metalloproteinase inhibitor 3 (TIMP3) and tropomyosin α3.

**Table 3:** Differentially expressed proteins between CON, LPS and TGF+LPS analyzed by LC-MS/MS- based proteomics.

Protein	Gene ID	Mr (kDa)	Quantified levels in ratio with ref samples ^1^			P-value
			CON	LPS	TGF+LPS	
**Stressed-response proteins**						
Heat shock cognate 71 kDa protein (HSPA8)	gi|345441750	71.1	0.78 ± 0.08 ^a^	1.05 ± 0.10 ^ab^	1.17 ± 0.03 ^b^	0.027
Glucose-regulated protein 58 kDa (GRP58)	gi|304365428	57.4	0.98 ± 0.03 ^ab^	0.85 ± 0.05 ^a^	0.99 ± 0.01 ^b^	0.040
Protein disulfide isomerase (PDI)	gi|358009193	56.9	1.03 ± 0.02 ^a^	0.89 ± 0.02 ^b^	1.01± 0.02 ^a^	0.004
Cyclophilin A (CYPA)	P62936	17.9	0.90 ± 0.03 ^a^	1.26 ± 0.08 ^b^	1.18 ± 0.10 ^ab^	0.043
Nucleolin	gi|335309939	78.2	0.88 ± 0.09 ^a^	1.14 ± 0.01 ^b^	1.19 ± 0.02 ^b^	0.015
**Protein related to metabolism, processing and DNA binding**						
Elongation factor 1-α1 (EEF1A1)	gi|4503471	50.5	0.80 ± 0.05 ^a^	1.20 ± 0.07 ^b^	1.09 ± 0.03 ^b^	0.004
Heterogeneous nuclear ribonucleoprotein U	gi|350589336	89.8	0.77 ± 0.10 ^a^	1.00 ± 0.06 ^ab^	0.84 ± 0.02 ^ab^	0.042
60S ribosomal protein L4	gi|335280113	48.4	0.97 ± 0.03 ^a^	1.26 ± 0.01 ^b^	1.22 ± 0.04 ^b^	0.007
Pyruvate kinase isozymes M1/M2 isoform 1	gi|194038728	59.6	0.82 ± 0.02 ^a^	0.97 ± 0.08 ^ab^	1.05 ± 0.01 ^b^	0.040
**Cytoskeleton and cell mobility**						
Tropomyosin alpha-3 chain isoform 2	gi|24119203	32.5	1.00 ± 0.01 ^a^	0.95 ± 0.02 ^a^	1.27 ± 0.06 ^b^	0.016
Filamin-B-like	gi|350591286	193.5	0.66 ± 0.10 ^a^	1.04 ± 0.09 ^b^	1.20 ± 0.05 ^b^	0.009
Myosin-9	gi|350583843	210.4	0.83 ± 0.04 ^a^	1.05 ± 0.04 ^b^	1.21 ± 0.02 ^b^	<0.001
Myosin light chain 3	P60662	16.9	0.84 ± 0.12 ^a^	1.16 ± 0.10 ^ab^	1.45 ± 0.17 ^b^	0.047
**Metal ion binding proteins**						
Protein S100-A6	Q2EN75	10.06	0.72 ± 0.01 ^a^	1.06 ± 0.07 ^b^	1.09 ± 0.04 ^b^	0.009
Metalloproteinase inhibitor 3 precursor (TIMP3)	gi|261244950	26.7	0.63 ± 0.03 ^a^	3.38 ± 0.91 ^b^	0.78 ± 0.22 ^a^	0.026

^1^ Values not sharing the same letters are significantly different.

**Fig 3:**
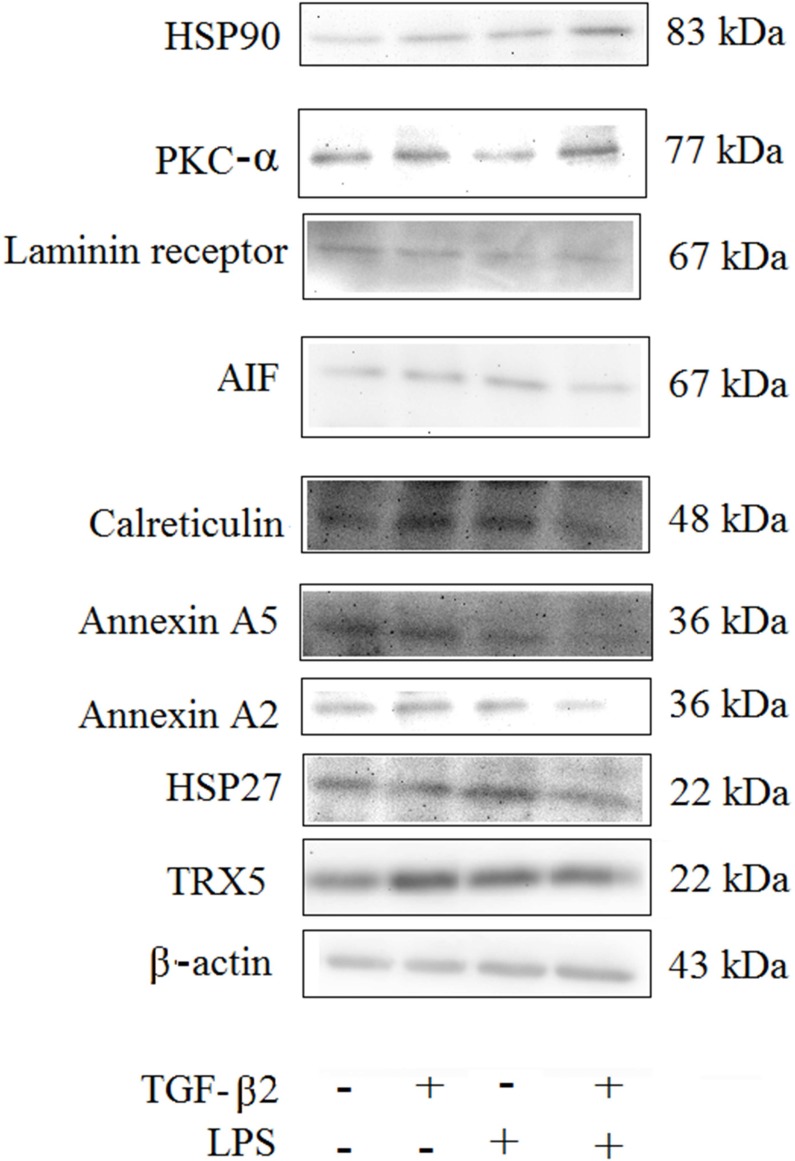
Western blot of stress-response proteins to support iTRAQ-LC-MS/MS-based proteomics. PsIc1 cells were treated with TGF-β2 and/or LPS for 24 h (n = 3 in each treatment). Only Annexin A2, Calreticulin, AIF, PKC-α and HSP90 were differentially expressed between treatments.

Nine additional proteins known to be associated with stress response selected based on previous literature were analyzed by Western blot (Fig. 3). After densitometry analysis and statistics, five of these proteins including annexin A2, AIF, calreticulin, HSP90 and PKC-α were differentially expressed among the treatment groups.

**Fig 4:**
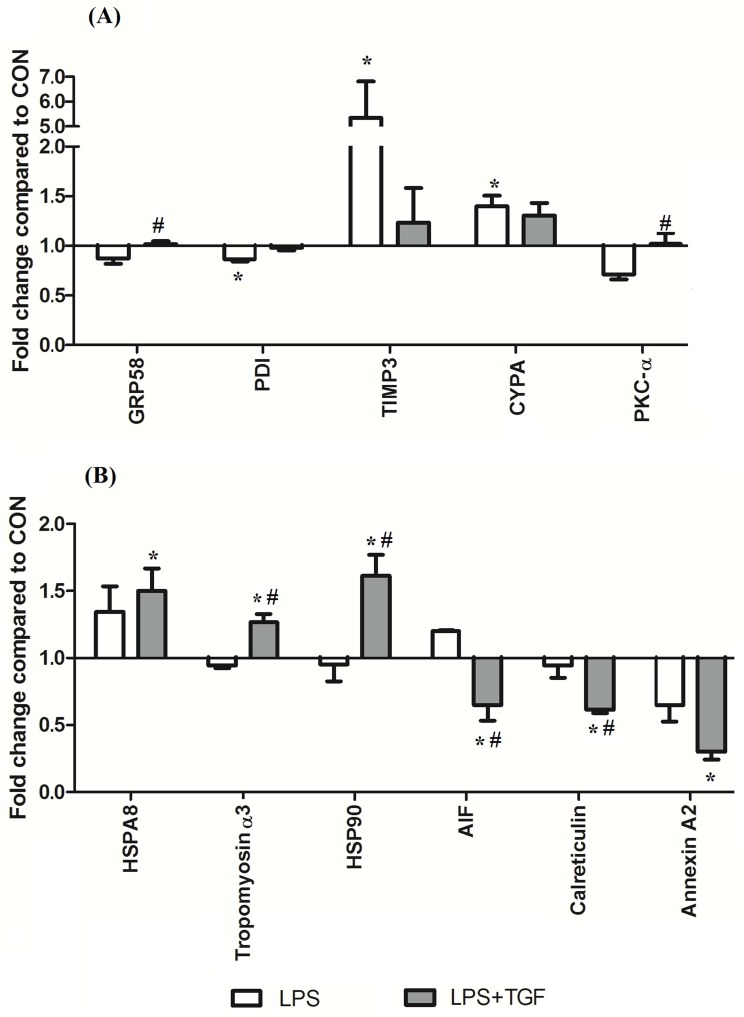
Differential patterns of stress-response proteins. Stress-response proteins regulated by TGF-β2 in LPS-challenged IECs were analyzed by both LC-MS/MS-based proteomics and Western blot. (A): TGF-β2 restored the levels of proteins up/down-regulated by LPS to their basal levels. (B): In LPS-challenged IECs, TGF-β2 altered the expression of proteins that were not affected by LPS alone. Values were means ± SEM (n = 3 in each treatment). *, #: P < 0.05, compared with CON and LPS, respectively.

Among the 13 identified proteins associated with stress response analyzed by both LC-MS proteomics (8 proteins) and Western blot (5 proteins), 11 of these followed two different patterns of regulation by both TGF-β2 and LPS (Fig. 4). In one group (Fig. 4A), the levels of GRP58, PDI, TIMP3, CYPA, and PKC-α were altered by LPS, and restored by TGF-β2 to those levels in CON. On the other hand, HSPA8, HSP90, AIF, annexin A2, calreticulin and tropomyosin α3 formed the other group (Fig. 4B) and these protein levels were altered only by TGF-β2 in the presence of LPS, but not by LPS alone. The stratification of these two protein groups based on differential patterns suggests distinct mechanisms for the TGF-β2-mediated protection of IECs against inflammatory stress.

### Protein-protein interaction network

**Fig 5:**
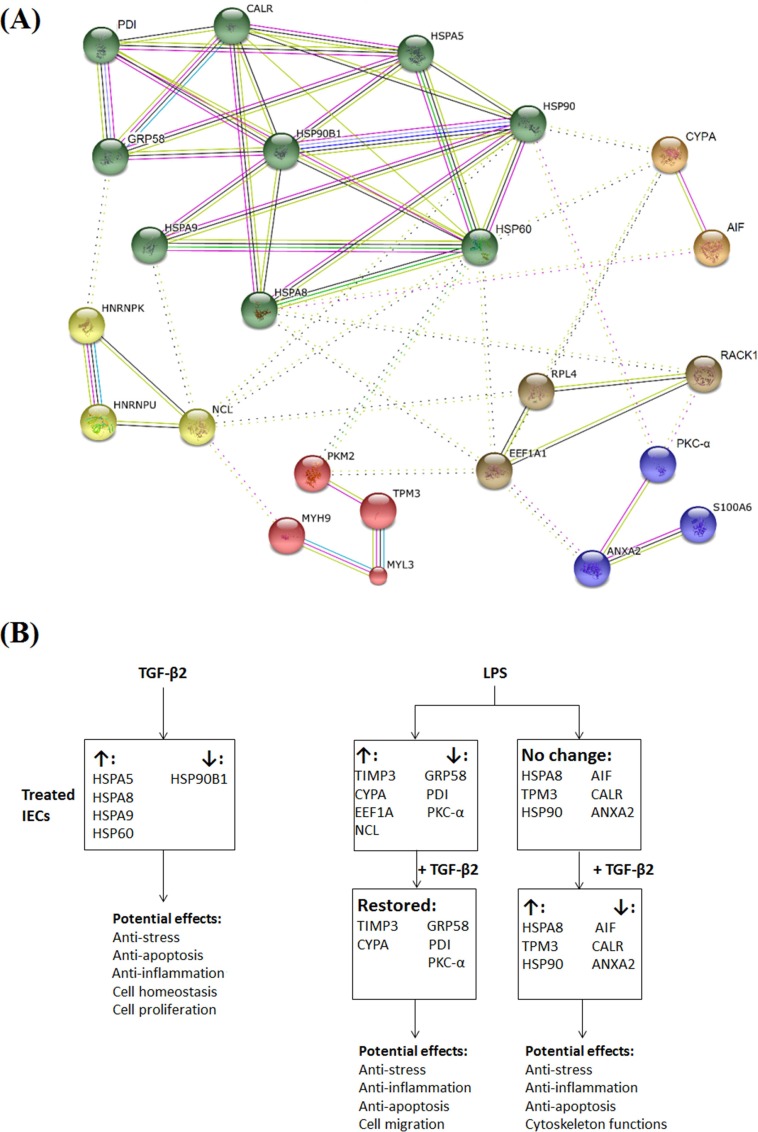
Protein interaction network and proposed protective mechanisms evoked by TGF-β2. (A) The protein-protein interaction network of the differentially expressed proteins regulated by TGF-β2 and LPS. Clusters of proteins are distinguished by colors of nodes formulated by STRING. Connecting lines show interactions based on co-expression (black), neighborhood (dark green), co-occurrence (blue), experiments (pink), database (aqua) and text-mining (light green). (B) Overview of stress-response proteins differentially expressed by TGF-β2 and LPS with their biological functions in relation to the potential effects of TGF-β2 in the immature intestine. ANXA2: annexin A2, CALR: calreticulin, HNRNPK: Heterogeneous nuclear ribonucleoprotein K, HNRNPU: Heterogeneous nuclear ribonucleoprotein U, MYH9: myosin 9, MYL3: Myosin light chain 3, NCL: nucleolin, PKM2: Pyruvate kinase isozymes M1/M2, RPL4: 60S ribosomal protein L4, S100A6: protein S100-A6, TPM3: tropomyosin α3.

All 30 differentially expressed proteins were further analyzed by the protein-protein interaction predictive tools in STRING. STRING assigns proteins into interaction clusters based on co-expression, neighborhood, co-occurrence, experiments, database and text mining data. Twenty-four proteins were assigned to six clusters (Fig. 5A). Nine HSPs were grouped in one single cluster. Within this cluster, HSP90B1 was the central molecule connecting the remaining eight proteins in the HSP cluster. In addition, each of five other clusters contained at least one stress-associated protein connecting to proteins in the HSP cluster, illustrated by dashed lines. Within the HSP cluster, HSP90, HSP60 and HSPA8 appeared to be the most tightly connected to proteins from the remaining five clusters. In addition, the stress response proteins differentially expressed by TGF-β2 and LPS are summarized in Fig. 5B together with their biological functions in relation to the potential effects of TGF-β2 in the immature intestine.

## Discussion

TGF-β2 is involved in different processes including cell proliferation and differentiation, and also protects the immature intestine against inflammation [3,6]. However, little is known about the molecular mechanisms underlying the effects of TGF-β2 in IECs, which may help to explain how TGF-β2 improves intestinal development and protects the intestine against stress such as enteral feeding and endotoxin challenge in newborns. The exposure of the immature intestine to endotoxin, such as LPS, activates TLR-4 signaling, promotes cellular stress, inflammation and intestinal injury [21,22], and may eventually lead to NEC, as observed in mice [8]. In the present study, we show for the first time that TGF-β2 indeed alters the levels of stress-response proteins in IECs. Moreover, TGF-β2 modulates the proteome of LPS-challenged IECs with most of the differentially expressed proteins associated with stress response. These changes may explain the regulatory effects of TGF-β2 in porcine IECs as well as in the porcine immature intestine.

### TGF-β2 regulates stress-response proteins

Five of the 13 proteins altered in TGF-β2-treated cells are HSPs, conserved molecular chaperones of misfolded proteins [23], including HSP60, HSPA5, HSPA8, HSPA9 and HSP90B1. HSPs are typically elevated under stress [23] to protect enterocytes and facilitate intestinal adaptation to gut colonization [12] or to regulate stress responses of the endoplasmic reticulum (ER) for maintaining cellular homeostasis [24]. HSP60, HSPA5, HSPA8 and HSPA9 levels were augmented by TGF-β2, suggesting enhanced cytoprotection by TGF-β2 in enterocytes. These mechanisms may be involved in the protective effects of TGF-β2 against intestinal diseases such as NEC.

Increased intestinal HSP70s including HSPA5, HSPA8 and HSPA9 play important roles in the control of cell growth, inhibition of apoptosis, protection against dietary stress, and degradation of TLR-4 thereby preventing NEC development in mice [8,11]. Elevated levels of these HSP70s by TGF-β2 in our study were consistent with that in the intestine of NEC-resistant pigs, such as parenterally fed preterm pigs, germ-free preterm pigs, and term pigs [11,12,14]. For instance, HSPA9 was up-regulated in the intestine of preterm pigs not receiving enteral formula, compared with formula-fed pigs [11]. HSPA5 and HSPA8 levels were increased in germ-free pigs, compared with pigs reared under conventional conditions [12]. These studies indicate increased protection against intestinal damage and probable increased degradation of surface TLR-4, thus preventing LPS-mediated inflammation in NEC-resistant pigs, via elevated HSP70s levels. HSPA8 showed higher expression in the intestine of term versus preterm pigs [14], suggesting their increased resistance to NEC. HSPA5 showed higher expression in healthy than NEC-affected intestinal specimens of infants [25], and elevated HSPA5 also protected mice against colitis [26].

In contrast to HSP70s, HSP90B1, one of the most abundant ER proteins [27], was down-regulated 2.5 fold by TGF-β2. This observation is consistent with what was observed in NEC-resistant preterm pigs subjected to oral antibiotic treatment [13]. The finding in our study suggests anti-inflammatory effects of TGF-β2 as HSP90B1-deficient mice lose TLR-4, thereby being more resistant to endotoxin shock [28].

HSPs also participate in TGF-β signaling and related pathways which may be involved in the protective effects of TGF-β2. HSPA8 binds to the orphan transcriptional activator MSG1 to suppress MSG1-induced Smad-mediated transcription, a pathway activated by TGF-βs [29]. HSPA5 enhances cell proliferation, apoptosis resistance and increases protection against stress and oxidative damage [30,31]. Additionally, HSP60 expressed on the cell surface can act as a co-receptor of CD14 to facilitate LPS binding and TLR-4 signaling [23]. HSP60 elevated by TGF-β2 in our study may be partly expressed on the cell surface, thereby regulating LPS binding and NF-κB signaling to stimulate physiological IL-8 secretion [32], which is crucial in attracting neutrophils for pathogenic clearance during the early phase of inflammation.

According to our knowledge, this is the first study demonstrating that TGF-β2 up-regulates HSP70s and HSP60 and down-regulates HSP90B1 in IECs. TGF-β1, another isomer of TGF-β, up-regulates HSP70s at both protein and mRNA levels [33–35] via posttranscriptional mechanisms by accelerating RNA processing and transport in chicken embryo cells without changing the rate of transcriptional activities [34]. This mechanism may also occur in TGF-β2-treated IECs in this study.

In addition to HSPs, other identified proteins were also involved in TGF-β and NF-κB signaling, as well as events related to intestinal protection such as proliferation and migration. Decreased G5PR levels may enhance physiological NF-κB activation and potentiate TGF-β signaling [36]. TGF-β2-mediated decrease in the levels of RACK1, a protein interacting with the TGF-β2 signaling molecule Smad3, may play an important role in IEC migration and wound healing as RACK1 inhibits cell migration [37]. ZNF268, GH1, hnRNPK, KRT1 and KATNAL2 are also involved in cell viability, migration, proliferation and differentiation [38–42]. These proteins, altered by TGF-β2, may reflect the mechanisms of TGF-β2-mediated cellular protection.

### Stress-response proteins regulated by TGF-β2 in LPS-challenged IECs

Two protein groups with two different patterns of regulation by both TGF-β2 and LPS may reflect the distinct mechanisms of TGF-β2 protection in IECs under stress conditions such as LPS stimulation. Group 1 (Fig. 4A), including GRP58, PDI, TIMP3, CYPA and PKC-α, were regulated by LPS but were restored to control levels by TGF-β2 in LPS-challenged IECs. Levels of proteins in group 2 (Fig. 4B), comprising HSPA8, HSP90, AIF, annexin A2, calreticulin and tropomyosin α3, were not affected by LPS but were altered by TGF-β2 in LPS-challenged IECs.

Within group 1, GRP58 and PDI function in adaptive response to protein misfolding and aggregation caused by ER stress [43]. Both of them act as catalysts of thiol-disulfide oxidation, and their inhibition prevents cell migration [44,45]. LPS-induced GRP58 and PDI down-regulation in this study is consistent with findings in RAW264.7 cells and monocytes treated by LPS [45,46]. This reduction may reflect the degradation of these proteins induced by oxidative stress of LPS, and this may result in inflammation as previously suggested [46]. The LPS-induced decrease in cell migration may also be via reducing GRP58 and PDI [44]. TGF-β2 treatment returned GRP58 and PDI to basal levels in LPS-challenged cells, suggesting that TGF-β2 exerts anti-inflammatory effects and maintains the physiological cell migration of IECs. TIMP3 is a protein associated with the extracellular matrix and inhibits metalloproteinase activity [47]. TIMP3 is up-regulated by cellular stressors in a similar way as LPS up-regulated TIMP3 in our study, and it has been suggested as a main modulator of several apoptosis pathways, such as Fas-mediated or TNF-related apoptosis [47–49]. In LPS-challenged cells, TGF-β2 restored TIMP3 expression to control levels, indicating protective effects of TGF-β2 against cellular stress and cell death. Similar to HSPs, the cyclophilin CYPA is a chaperone protecting cells against stresses, and is also involved in cell migration [50]. LPS up-regulated CYPA, suggests an increased requirement for this protein during inflammation. The presence of TGF-β2 may be sufficient for cellular protection, thus TGF-β2 restored CYPA in LPS-challenged IECs to control levels. This effect reflects the impact of TGF-β2 on balancing cellular homeostasis to counteract stress responses.

Group 2 included proteins altered only by TGF-β2 in LPS-challenged IECs, emphasizing the importance of TGF-β2 in regulating proteins that protect IECs exposed to LPS. HSPA8 overexpression reduced LPS-induced production of IL-6 and TNF-α in rats [21]. In the present study, TGF-β2 stimulated LPS-challenged IECs to elevate HSPA8, likely reflecting the protective effects of HSPA8 in inhibition of pro-inflammatory cytokines, as our previous findings showed that TGF-β2 suppressed LPS-induced IL-6, IL-1β and TNF-α secretion in PsIc1 cells [15]. On the other hand, tropomyosin α3 interacts with HSP27, an HSP involved in cytoskeleton reorganization and contraction of colon smooth muscle cells [51,52]. The elevation of tropomyosin α3 by TGF-β2 in LPS-challenged IECs proposes that TGF-β2 may stabilize the cytoskeleton in response to LPS via its interaction with HSP27.

Based on a literature review, we selected nine additional proteins associated with stress responses not observed in the proteomic data for Western blot analysis. Five of them showed differential patterns between treatments. PKC-α, which is involved in intestinal cell maturation and proliferation [53], was restored to the levels in CON by TGF-β2 in LPS-challenged cells. This indicates that TGF-β2 may reverse LPS-inhibited proliferation. The remaining four proteins AIF, HSP90, annexin A2 and calreticulin (group 2) were differentially expressed only by TGF-β2 in LPS-challenged IECs. Calreticulin is involved in folding of nascent proteins and its overexpression protects cells against oxidative stress [54]. HSP90 plays important roles via co-localization with nucleolin to protect cells against apoptosis [55]. Apoptosis induced by AIF, the death effector released from mitochondria during the early phase of apoptosis [56], is inhibited by HSPA8 via prevention of AIF translocation [56]. LPS triggered a tendency of elevated AIF levels in our study, similar to what occurred in LPS-induced cell death as previously reported [57]. TGF-β2 markedly decreased AIF levels in LPS-challenged IECs, implying that TGF-β2 may attenuate LPS-induced apoptosis. Reduced AIF is also related to the increase of HSPA8 in the TGF+LPS group, suggesting a role for elevated HSPA8 to antagonize AIF [56]. Annexin A2 is an HSP27-interacting protein that protects cells against UV irradiation [58] and facilitates cell migration [59]. Reduced annexin A2 by TGF+LPS may be an important stage prior to cellular protection, cell migration and wound healing.

The protein-protein interaction network (Fig. 5A), which illustrates the close interaction between all differentially expressed proteins, demonstrates strong interactions among HSPs, and between HSPs and other stress-response proteins. This suggests that the regulatory effects of TGF-β2 in LPS-challenged IECs may be mediated either directly or indirectly by the functions of HSPs, which are associated with NEC progression in both mice and pig models [8,10]. We also proposed the overall summary of how TGF-β2 protects naïve IECs and inflamed IECs (Fig. 5B). TGF-β2 alone altered HSPs resulting in effects such as anti-stress, anti-apoptosis, anti-inflammation, cell proliferation and homeostasis. These effects suggest that TGF-β2 assists naïve IECs to be prepared for potential stresses. Indeed, when LPS-challenged cells were treated with TGF-β2, the functions of modulated stress-response proteins confirmed the protective effects exerted by TGF-β2 alone.

In conclusion, multiple HSPs and stress-response proteins were differentially expressed by TGF-β2 in porcine IECs and LPS-challenged IECs. These proteins also play roles in TGF-β and TLR-4 signaling pathways and in intestinal disease pathogenesis. The biological functions of these proteins suggest that TGF-β2 is a regulator of cellular homeostasis to protect IECs against inflammatory stimuli, such as LPS. As LPS-TLR-4 signaling, as well as TLR-4 and HSP interactions are crucial elements in intestinal inflammation [8], our data support the notion that TGF-β2 may protect against intestinal diseases during early life. In the future, the effects of TGF-β2-supplemented formulas on LPS-induced intestinal damage should be tested in *in-vivo* models, such as the NEC- and sepsis-sensitive preterm pig. This further work would pave the way for testing TGF-β2 effects on intestinal health and against sepsis in preterm infants.

## Supporting Information

S1 Table All identified proteins in CON, LPS and TGF+LPS used for statistical analysis with P-values. (PDF)
