# Supplementary material for: Protective Effects of Transforming Growth Factor β2 in Intestinal Epithelial Cells by Regulation of Proteins Associated with Stress and Endotoxin Responses
Source: PLoS One. 2015 Feb 10;10(2):e0117608. doi: 10.1371/journal.pone.0117608 (PMC4323210; doi:10.1371/journal.pone.0117608)
Supplement: S1 Table — (PDF) [file pone.0117608.s001.pdf]

Table S1. All identified proteins in CON, LPS and TGF+LPS used for statistical analysis with P-values.

Accession Nr	Name	Mr (kDa)	CON			LPS			TGF+LPS			P-values			
			Plex 1- 115/114	Plex 2-115/114	Plex 3-115/114	Plex 1- 117/114	Plex 4- 116/114	Plex 3- 116/114	Plex 4- 117/114	Plex 2- 117/114	Plex 3- 117/114	ANOVA	CON vs. LPS	CON vs. TGF+LPS	LPS vs. TGF+LPS
gi|4504523	10 kDa heat shock protein, mitochondrial [Homo sapiens]	10.925	1.137	1	1.005	1.385	0.945	1.061	1.123	1.082	1.079	0.7750	0.7575	0.9110	0.9480
gi|4507953	14-3-3 protein zeta/delta [Homo sapiens]	27.899	0.805		0.574	1.022	1.035	1.075	0.783		0.995	0.0631	0.0550	0.2882	0.3784
P62844	40S ribosomal protein S15	17.04	0.795			1.005	1.283		1.178			0.5315	0.5489	0.5684	0.9891
gi|45268973	40S ribosomal protein S3A [Sus scrofa]	-		1.027	0.748	0.448		1.222		1.227	1.481	0.3877	0.9878	0.4739	0.4148
gi|359811347	60 kDa heat shock protein, mitochondrial [Sus scrofa]	61.114	0.981	1.116	1.153	0.956	1.003	0.952	1.081	1.019	1.016	0.1372	0.1225	0.6420	0.3876
A1XQU3	60S ribosomal protein L14	23.33	0.923	0.766	1.048	1.527	0.912	1.266	1.052	1.058	1.234	0.2295	0.2111	0.4904	0.7618
P62831	60S ribosomal protein L23	14.865	0.76	0.883	0.848	1.076	1.186	0.79	1.1	1.155	0.86	0.2641	0.3576	0.2930	0.9846
Q95281	60S ribosomal protein L29	17.498		0.825	1.003		1.179	0.946	1.368	1.084	1.499	0.1527	0.7147	0.1470	0.3706
gi|4506631	60S ribosomal protein L30 [Homo sapiens]	12.989		1.129	0.744		1.006	0.939	1.053	1.316	1.012	0.5102	0.9785	0.5354	0.6489
P62901	60S ribosomal protein L31	14.463		1.164	0.887	1.281	1.17	1.276	0.968	1.409	1.238	0.4159	0.4104	0.5255	0.9613
gi|15431303	60S ribosomal protein L9 [Homo sapiens]	21.992		0.672	0.905		0.638	1.135		0.704	1.185	0.8747	0.9432	0.8661	0.9795
Q6QRN9	ADP/ATP translocase 3	32.91		0.794	0.933		0.758	0.961	0.727	0.845	1.033	0.9975	0.9995	0.9992	0.9974
gi|345441771	aldolase C, fructose-bisphosphate [Sus scrofa]	39.849		0.831	0.817		0.95	1.261	1.093	1.242	1.578	0.1467	0.4402	0.1308	0.5880
gi|340007404	alpha-actinin-1 [Sus scrofa]	103.251	0.562	0.827	0.812	1.07	1.077	1.394	0.959	0.955	1.444	0.0823	0.0931	0.1454	0.9349
P19620	Annexin A2	38.534	0.759	1.091	0.886	0.88	1.127	0.996	1.069	1.214	1.156	0.1547	0.6851	0.1399	0.3998
gi|21636588	ATP synthase gamma subunit 1 [Sus scrofa]	4.953		0.97	1.028		1.117	1.047	1.045	0.774	0.891	0.2622	0.7121	0.5967	0.2442
gi|297591975	ATP synthase subunit alpha, mitochondrial [Sus scrofa]	59.793	0.904	0.947	1.018	1.053	0.908	0.967	0.978	0.927	0.92	0.7671	0.9066	0.9466	0.7491
Q29307	ATPase inhibitor, mitochondrial	12.184		0.922	0.888		0.996	1.163	1.294	1.18	0.97	0.2377	0.4511	0.2195	0.8406
gi|342349319	calnexin precursor [Sus scrofa]	68.107		0.726	0.819		0.686	0.712	0.915	1.027	0.781	0.1477	0.7302	0.3507	0.1437
P28491	Calreticulin	48.288		0.984	0.856		0.856	1.059	1.007	1.049	1.067	0.3765	0.9060	0.3766	0.5914
gi|230338	Chain E, Structure Of The Trypsin-Binding Domain	23.975	0.97	0.836	0.795	1.075	1.015	0.969	0.991	0.959	0.873	0.0977	0.0843	0.4527	0.4142
Q5S3G4	Cytochrome c oxidase subunit 5B, mitochondrial	13.783		1.216	1.192		1.126	0.945	1.104	1.169	0.851	0.4258	0.4908	0.4579	0.9988
Q9N0F1	Dihydrolipoyllysine-residue succinyltransferase component of 2-oxoglutarate dehydrogenase complex	48.977	0.916	0.796	1.232	0.797	1.177	0.88	1.164	0.902	1.361	0.5503	0.9846	0.6593	0.5653
gi|4503471	elongation factor 1-alpha 1 [Homo sapiens]	50.535	0.899	0.765	0.733	1.329	1.122	1.138	1.157	1.074	1.051	0.0040	0.0038	0.0161	0.3997
P00355	Glyceraldehyde-3-phosphate dehydrogenase	35.836	0.858	0.863	0.928	1.26		0.978		1.175	1.092	0.0978	0.1494	0.1288	0.9900
gi|345441750	heat shock 70kDa protein 8 [Sus scrofa]	71.106	0.611	0.847	0.875	0.873	1.055	1.206	1.131	1.15	1.222	0.0270	0.1014	0.0242	0.5195
Q5S1U1	Heat shock protein beta-1	22.942	1.039	0.816	0.773	1.337	1.115	1.07	1.115	1.257	1.061	0.0580	0.0701	0.0995	0.9585
gi|4504445	heterogeneous nuclear ribonucleoprotein A1 isoform a [Homo sapiens]	35.098	1.054		0.929	0.941	0.823	0.995	0.895		0.939	0.5797	0.6091	0.6378	0.9992
gi|343887440	heterogeneous nuclear ribonucleoprotein C (C1/C2) isoform 1 [Sus scrofa]	34.371	0.692	1.016	1.215	0.62	0.975	0.907	0.964	0.929	0.959	0.6400	0.6507	0.9870	0.7382
gi|346716298	heterogeneous nuclear ribonucleoprotein G [Sus scrofa]	42.249		0.894	1.182		0.863	1.066	0.961	0.982	1.153	0.8520	0.8722	0.9989	0.8706
gi|392513715	heterogeneous nuclear ribonucleoprotein K [Sus scrofa]	51.291		0.839	0.783		1.142	1.005	1.017	1.099	1.238	0.0518	0.1036	0.0510	0.8686
gi|349732227	heterogeneous nuclear ribonucleoprotein M [Sus scrofa]	73.868	1.057	1.014		0.858	0.907		0.986	1.224		0.2251	0.3992	0.7855	0.2156
gi|4504447	heterogeneous nuclear ribonucleoproteins A2/B1 isoform A2 [Homo sapiens]	36.041	1.053	0.963	0.947	0.999		1.023		1.169	0.944	0.7210	0.9566	0.7001	0.8716
gi|68534962	histone H1.2-like protein [Sus scrofa]	30.033	1.032	1.105	1.283	1.086	1.12	1.149	1.13	1.016	1.01	0.4728	0.9494	0.4691	0.6353
gi|72535198	histone H1.3-like protein [Sus scrofa]	31.336	1.024	1.103	1.249	1.106	1.168	1.104	1.139	1.012	1.008	0.4937	0.9999	0.5551	0.5495
gi|45479848	histone H1.3-like protein [Sus scrofa]	22.143	0.997	1.093	1.251	1.068	1.179	1.106	1.15	1.006	1.006	0.6684	0.9985	0.7297	0.7004
gi|4504239	histone H2A type 1 [Homo sapiens]	14.083	1.019	1.062	1.056	1.07	1.028	1.179	0.988	1.042	1.08	0.4489	0.5713	0.9774	0.4638
gi|119331192	histone H2A.x [Bos taurus]	17.137	1.187	1.072	1.078	1.088	1.02	1.164	1.02	1.048	1.065	0.3925	0.8911	0.3767	0.6102
gi|10800138	histone H2B type 1-D [Homo sapiens]	13.928	0.996	1.091	1.186	0.978		0.952		0.931	0.942	0.1213	0.2197	0.1371	0.9094
gi|18105048	histone H2B type 1-K [Homo sapiens]	16.908		1.092	1.188			0.955		0.934	0.944	0.0917	0.1566	0.0948	0.9608
gi|4504261	histone H2B type 1-N [Homo sapiens]	16.94	0.997	1.093	1.188	0.981		0.956		0.933	0.942	0.1231	0.2283	0.1369	0.8950
gi|4504277	histone H2B type 2-E [Homo sapiens]	13.912	0.996		1.188	0.989	1.022	0.948	0.925		0.941	0.1990	0.3496	0.1887	0.7223
gi|4504281	histone H3.1 [Homo sapiens]	17.412	1.036	1.545	1.241	0.982	0.832	1.076	0.689	0.809	1.046	0.0843	0.2045	0.0815	0.7584
P62802	Histone H4	11.367	0.956	0.99	0.966	0.994	1.076	0.946	0.814	0.829	0.967	0.0863	0.7839	0.2002	0.0842
gi|345091038	keratin 74 [Sus scrofa]	63.383	0.935	0.792	0.996	0.906	1.075	1.151	1.1	0.938	1.124	0.2722	0.3533	0.3101	0.9933
gi|227430407	keratin, type II cytoskeletal 8 [Sus scrofa]	54.394	1.056	1.056	1.071	0.953	0.984	0.96	0.964	0.956	0.95	0.0001	0.0002	0.0001	0.6214
gi|350276172	leucine-rich repeat-containing protein 59 [Sus scrofa]	35.288		1.259	1.209		1.244	0.912	1.413	1.23	1.082	0.5640	0.6494	0.9986	0.5756
P00346	Malate dehydrogenase, mitochondrial	35.596	0.965		0.963	0.714	0.997	0.994	1.012		1.122	0.4123	0.8456	0.6960	0.3846

gi|261244950	metalloproteinase inhibitor 3 precursor [Ovis aries]	26.709		0.658	0.605		2.462	4.291	0.8	0.388	1.146	0.0264	0.0368	0.9716	0.0326
gi|7657257	mitochondrial import receptor subunit TOM20 homolog [Homo sapiens]	16.501	0.85	1.749	1.059		0.801	0.961	1.074	1.338	1.085	0.5193	0.5164	0.9765	0.6153
P60662	Myosin light polypeptide 6	16.93	0.682	0.767	1.076	0.981	1.197	1.313	1.481	1.144	1.73	0.0471	0.2736	0.0394	0.3402
P62936	Peptidyl-prolyl cis-trans isomerase A	17.869	0.935	0.831	0.936	1.283	1.103	1.391	0.987	1.195	1.345	0.0434	0.0436	0.1099	0.7506
gi|4505753	phosphoglycerate mutase 1 [Homo sapiens]	28.928		0.804	0.489		0.984	1.199	1.002	1.365	1.212	0.0724	0.1524	0.0685	0.8294
gi|41386798	polyadenylate-binding protein 1 [Bos taurus]	70.91		1.121	0.439		1.151	0.656	0.985	0.896	0.612	0.9320	0.9261	0.9842	0.9685
Q29099	Polypyrimidine tract-binding protein 1	59.855		0.916	0.966		0.971	1.035	0.967	1.096	1.173	0.2670	0.7308	0.2499	0.5889
gi|335310941	PREDICTED: 40S ribosomal protein S2-like [Sus scrofa]	24.442		1.015	0.87		1.162	0.973	0.973	0.98	1.078	0.4793	0.4519	0.7273	0.7935
gi|350579657	PREDICTED: 78 kDa glucose-regulated protein [Sus scrofa]	73.223	1.296	0.792	0.986	1.22	0.979	1.089	1.122	1.088	1.121	0.7952	0.8567	0.8021	0.9936
gi|335287187	PREDICTED: ATP synthase subunit b, mitochondrial [Sus scrofa]	28.68	0.988	0.974	1.013	0.706	0.883	0.824	0.717	0.817	1.237	0.4361	0.4141	0.8760	0.6770
gi|311248247	PREDICTED: ATP synthase subunit delta, mitochondrial-like isoform 1 [Sus scrofa]	17.464		0.706	1.278		0.928	1.199	1.011	0.916	1.08	0.9443	0.9492	0.9987	0.9552
gi|335306989	PREDICTED: ATP-dependent RNA helicase A-like [Sus scrofa]	143.363	1.453	1.137	1.614	1.148	0.942	1.462	0.862	1.087	1.156	0.2132	0.5026	0.1931	0.7094
gi|335306675	PREDICTED: B-cell receptor-associated protein 31-like [Sus scrofa]	27.913		0.785	1.025		0.956	1.072	1.062	1.03	1.112	0.3008	0.5601	0.2770	0.8277
gi|350582226	PREDICTED: bifunctional methylenetetrahydrofolate dehydrogenase/cyclohydrolase[Sus scrofa]	11.107	0.85		0.79	0.862	1.046	1.097	1.051		0.877	0.2858	0.2724	0.4562	0.9254
gi|311273514	PREDICTED: brain acid soluble protein 1-like isoform 1 [Sus scrofa]	23.074		0.806	0.857		1.066	1.26	1.048	0.876	1.1	0.0911	0.0802	0.2881	0.3603
gi|335282386	PREDICTED: elongation factor 2 [Sus scrofa]	96.501		0.797	0.677		0.969	0.858	0.806	1.014	0.944	0.1868	0.2611	0.1973	0.9955
gi|350591286	PREDICTED: filamin-B-like [Sus scrofa]	193.524	0.481	0.687	0.816	0.888	1.06	1.183	1.107	1.293	1.194	0.0087	0.0360	0.0082	0.4235
gi|311275457	PREDICTED: filamin-C isoform 1 [Sus scrofa]	293.428	0.483	0.618	0.918	0.883	1.134	1.204	0.986	0.863	1.213	0.0825	0.0935	0.1445	0.9390
gi|350589336	PREDICTED: heterogeneous nuclear ribonucleoprotein U [Sus scrofa]	89.814	0.848	0.574	0.888	1.105	0.97	0.912	0.881	0.82	0.807	0.1259	0.1194	0.7749	0.2865
gi|350590319	PREDICTED: keratin, type I cytoskeletal 17-like [Sus scrofa]	49.244		1.078	1.006		0.906	0.96	0.907	0.867	1.005	0.1899	0.2704	0.1981	0.9917
gi|350584002	PREDICTED: keratin, type I cytoskeletal 18 [Sus scrofa]	44.096	1.067	1.151	1.056	0.909	0.887	0.942	0.894	0.929	0.951	0.0020	0.0025	0.0037	0.9212
gi|311267276	PREDICTED: keratin, type I cytoskeletal 19-like [Sus scrofa]	44.186	1.041	1.13	1.069	0.896	0.941	0.929	0.892	0.891	0.946	0.0016	0.0033	0.0023	0.9023
gi|311255377	PREDICTED: keratin, type II cuticular Hb5 [Sus scrofa]	59.529		1.192	1.247		0.982	1.101	1.03	1.09	1.083	0.0477	0.0559	0.0686	0.8503
gi|350583970	PREDICTED: keratin, type II cytoskeletal 75-like [Sus scrofa]	59.901	1.107	0.784	0.889	0.967	1.24	1.205	1.232	1.044	1.17	0.1706	0.2333	0.2058	0.9946
gi|311255413	PREDICTED: keratin, type II cytoskeletal 79 [Sus scrofa]	58.15		0.842	0.874		1.207	1.149	1.156	0.934	1.168	0.0629	0.0613	0.1229	0.5889
gi|335283403	PREDICTED: lamin-B1 [Sus scrofa]	66.73	1.074	1.012	1.379	0.866	0.896	1.002	0.998	0.682	1.431	0.5490	0.5204	0.8346	0.8403
gi|335280113	PREDICTED: LOW QUALITY PROTEIN: 60S ribosomal protein L4 [Sus scrofa]	48.355		0.945	0.999		1.264	1.245	1.243	1.145	1.257	0.0073	0.0089	0.0114	0.6641
gi|350582175	PREDICTED: mitochondrial inner membrane protein, partial [Sus scrofa]	81.561		1.249	1.212		0.799	0.962	0.693	0.805	1.192	0.2274	0.2796	0.2571	0.9954
gi|311268173	PREDICTED: myb-binding protein 1A [Sus scrofa]	152.56		1.004	1.24		0.73	0.818	0.766	1.056	1.088	0.1910	0.1729	0.5715	0.4240
gi|350583843	PREDICTED: myosin-9 [Sus scrofa]	210.417	0.82	0.769	0.895	1.115	0.991	1.029	1.161	1.233	1.232	0.0006	0.0080	0.0006	0.0294
gi|74007632	PREDICTED: non-POU domain-containing octamer-binding protein isoform 2 [Canis lupus familiaris]	54.211	0.622		0.941	0.962	0.986	0.761	0.922		0.845	0.6672	0.6595	0.7748	0.9881
gi|311271760	PREDICTED: nucleolar and coiled-body phosphoprotein 1 [Sus scrofa]	90.022	1.288	0.858	0.57	1.102	0.92	1.177	1.097	1.175	0.929	0.6425	0.6919	0.6900	1.0000
gi|335309939	PREDICTED: nucleolin-like [Sus scrofa]	78.252	0.696	0.938	0.997	1.127	1.122	1.159	1.193	1.148	1.219	0.0151	0.0359	0.0171	0.7980
gi|311273930	PREDICTED: nucleophosmin-like isoform 1 [Sus scrofa]	37.482		0.898	0.98		1.102	1.449	1.131	1.087	1.09	0.1324	0.1175	0.4185	0.3865
gi|311259195	PREDICTED: plasminogen activator inhibitor 1 RNA-binding protein-like isoform 2 [Sus scrofa]	44.242		0.628	0.828		0.956	1.138	0.889	1.171	1.393	0.1819	0.3536	0.1685	0.8456
gi|194038728	PREDICTED: pyruvate kinase isozymes M1/M2 isoform 1 [Sus scrofa]	-		0.799	0.847		1.048	0.884	1.055	1.033	1.046	0.0403	0.1586	0.0355	0.4161
gi|350594669	PREDICTED: ribosome-binding protein 1 [Sus scrofa]	159.236		1.33	0.85		0.888	1.104	1.064	1.082	1.003	0.8853	0.8754	0.9703	0.9483
gi|335303222	PREDICTED: STE20-related kinase adapter protein beta [Sus scrofa]	47.327	1.883	1.395	1.008	0.958	0.866	0.754	0.602	0.826	0.837	0.0460	0.0931	0.0515	0.8867
gi|311250237	PREDICTED: stress-70 protein, mitochondrial [Sus scrofa]	73.96	0.983	1.015	0.997	0.902	0.926	1.046	1.037	1.089	0.989	0.2656	0.6504	0.6547	0.2397
gi|335285948	PREDICTED: very long-chain specific acyl-CoA dehydrogenase, mitochondrial-like isoform 1 [Sus scrofa]	70.779		1.168	0.91		0.572	0.735	0.755	0.752	0.705	0.0492	0.0520	0.0824	0.7023

Q3ZD69	Prelamin-A/C	74.219	1.01	1.009	0.987	0.993		0.839		1.095	0.919	0.5097	0.5459	0.9976	0.5657
gi|343780941	prohibitin 2 [Sus scrofa]	33.367	1.129	0.665	0.908	1.005	0.994	0.794	0.877	0.804	0.675	0.5428	0.9715	0.6749	0.5464
gi|358009193	prolyl 4-hydroxylase beta polypeptide [Sus scrofa]	56.861	0.994	1.035	1.048	0.875	0.865	0.92	0.984	1.05	0.985	0.0036	0.0043	0.7511	0.0090
gi|304365428	protein disulfide-isomerase A3 precursor [Sus scrofa]	57.391	0.926	0.984	1.015	0.901	0.895	0.755	0.969	1.01	0.994	0.0404	0.0750	0.9347	0.0487
Q2EN75	Protein S100-A6	10.06		0.735	0.712		1.133	0.988	1.099	1.143	1.022	0.0086	0.0168	0.0085	0.8987
gi|343403779	ribosomal protein L13 [Sus scrofa]	24.433		0.915	0.968		0.71	0.997	0.903	1.072	0.918	0.6336	0.7665	0.9774	0.6195
gi|24119203	tropomyosin alpha-3 chain isoform 2 [Homo sapiens]	32.473		0.988	1.014		0.961	0.929	1.152	1.344	1.309	0.0156	0.7446	0.0358	0.0187
P63053	Ubiquitin-60S ribosomal protein L40	14.728	1.007		0.83	0.913	0.959	0.817	1.219		0.849	0.6332	0.9864	0.7488	0.6249

Search Parameters

<u>Subsamples: Plex 1, Plex 2, Plex 3, Plex 4</u>

Database: 1::NCBInr 2::SwissProt
Fasta Version: 1::NCBInr_20130902.fasta 2::SwissProt_2013_08.fasta
Sequences: 33374
Taxonomy: Sus scrofa (Pig)
Enzyme: Trypsin
Quantitation Protocol: iTRAQ 4plex
Fixed modifications: iTRAQ4plex (N-term),iTRAQ4plex (K)
Variable modifications: (M),iTRAQ4plex (Y)
Mass values: Monoisotopic
Peptide mass tolerance: 15 ppm
Fragment mass tolerance: 0.2 Da
Max missed cleavages: 1
Instrument type: ESI-QUAD-TOF
Ions score or expect cut-off: 0.005
Scoring : MudPIT

<u>Subsamples: Plex 4</u>

Significance threshold p 0.01
FDR 5.13%
MS/MS queries : 33625 (10% spectral use)

<u>Subsamples: Plex 3</u>

Significance threshold p 0.01
FDR 6.31%
MS/MS queries : 33281 (7.9% spectral use)

<u>Subsamples: Plex 1</u>

Significance threshold p 0.01
FDR 6.65%
MS/MS queries : 26112 (5.3% spectral use)

<u>Subsamples: Plex 2</u>

Significance threshold p 0.01
FDR 11.54%
MS/MS queries : 34241 (9.9% spectral use)

Quantitation Parameters

<u>Subsamples: Plex 1, Plex 2, Plex 3, Plex 4</u>

Protein ratio type: Weighted
Min. precursor charge: 1
Min. # peptides: 2
Unique peptides only: No
Normalisation: Sum
Outlier removal: Auto
Peptide threshold: At least homology
